# *trans*-Dichloridobis(2-methyl­aniline-κ*N*)palladium(II)

**DOI:** 10.1107/S1600536809008472

**Published:** 2009-03-14

**Authors:** V. Bon, A. Dudko, S. Orysyk, V. Pekhnyo

**Affiliations:** a V.I. Vernadskii Institute of General and Inorganic Chemistry, NAS Ukraine, Kyiv 03680, Ukraine

## Abstract

In the title compound, [PdCl_2_(C_7_H_9_N)_2_], the Pd atom is situated on an inversion centre and displays a distorted square-planar coordination environment. The crystal structure displays weak inter­molecular N—H⋯Cl hydrogen bonding.

## Related literature

For the cytostatic and anti­tumoral activity of Pd complexes with N*-*containing organic ligands, see: Casas *et al.* (2008 ▶); Curic *et al.* (1996 ▶). For related structures, see: Baldovino-Pantaleon *et al.* (2007 ▶); Navarro–Ranninger *et al.* (1987 ▶); Vogels *et al.* (1999 ▶). For bond-length data, see: Allen *et al.* (1987 ▶).
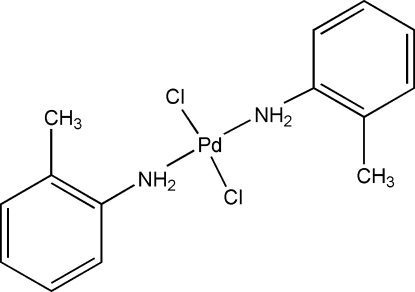


## Experimental

### 

#### Crystal data

[PdCl_2_(C_7_H_9_N)_2_]
*M* *_r_* = 391.60
Monoclinic, 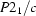

*a* = 12.1841 (3) Å
*b* = 8.0653 (2) Å
*c* = 7.5407 (2) Å
β = 97.346 (2)°
*V* = 734.93 (3) Å^3^
*Z* = 2
Mo *K*α radiation
μ = 1.61 mm^−1^
*T* = 173 K
0.17 × 0.16 × 0.04 mm

#### Data collection

Bruker APEXII CCD diffractometer
Absorption correction: multi-scan (*SADABS*; Bruker, 2005 ▶) *T*_min_ = 0.777, *T*_max_ = 0.932
8179 measured reflections
1502 independent reflections
1118 reflections with *I* > 2σ(*I*)
*R*_int_ = 0.074

#### Refinement

*R*[*F* ^2^ > 2σ(*F* ^2^)] = 0.034
*wR*(*F* ^2^) = 0.060
*S* = 1.03
1502 reflections
97 parameters
2 restraints
H atoms treated by a mixture of independent and constrained refinement
Δρ_max_ = 0.48 e Å^−3^
Δρ_min_ = −0.66 e Å^−3^

### 

Data collection: *APEX2* (Bruker, 2005 ▶); cell refinement: *SAINT* (Bruker, 2005 ▶); data reduction: *SAINT*; program(s) used to solve structure: *SHELXS97* (Sheldrick, 2008 ▶); program(s) used to refine structure: *SHELXL97* (Sheldrick, 2008 ▶); molecular graphics: *SHELXTL* (Sheldrick, 2008 ▶); software used to prepare material for publication: *publCIF* (Westrip, 2009 ▶).

## Supplementary Material

Crystal structure: contains datablocks I, global. DOI: 10.1107/S1600536809008472/rk2132sup1.cif

Structure factors: contains datablocks I. DOI: 10.1107/S1600536809008472/rk2132Isup2.hkl

Additional supplementary materials:  crystallographic information; 3D view; checkCIF report

## Figures and Tables

**Table 1:** Hydrogen-bond geometry (Å, °)

*D*—H⋯*A*	*D*—H	H⋯*A*	*D*⋯*A*	*D*—H⋯*A*
N1—H1*N*⋯Cl1^i^	0.85 (2)	2.71 (3)	3.410 (4)	141 (3)
N1—H2*N*⋯Cl1^ii^	0.90 (2)	2.43 (3)	3.319 (4)	172 (3)

Symmetry codes: (i) 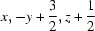; (ii) 
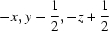
.

**Fig. 1.**
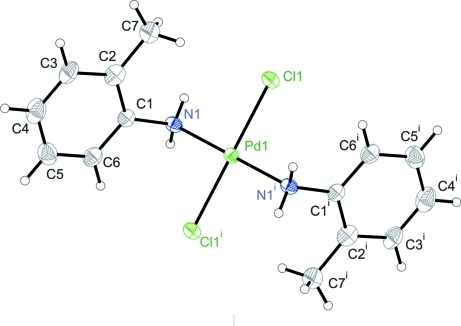
Molecular structure of the title compound with the atom numbering scheme. Displacement ellipsoids are drawn at 50% probability lervel. H atoms are presented as a small spheres of arbitrary radius. Symmetry code: (i) -x, 1-y, -z.

**Fig. 2.**
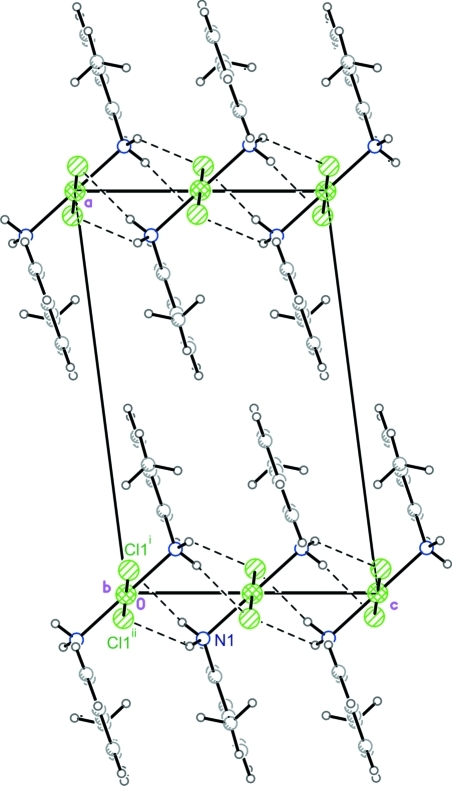
Crystal packing of title compound, projection along b axis. Dashed lines indicate hydrogen bonds [Symmetry code: (i) -x, y-1/2, 1/2-z; (ii) x, 3/2-y, 1/2+z].

## supplementary crystallographic information

### Comment

Palladium complex compounds with *N*–containing organic ligands attract constant scientific interest due to its cytostatic and antitumoral activity (Curic *et al.*, 1996; Casas *et al.*, 2008). Asymmetric unit of title compound contains half of a molecule, other one generates by the symmetry operator of inversion centre (Fig. 1). Pd atom shows a square–planar geometry of coordination environment, which contain two chlorine atoms in *trans*–position and a two amino groups of *o*–toluidine. Bond lengths and angles have normal values (Allen *et al.*, 1987). The crystal structure displays week intermolecular N—H···Cl hydrogen bonding (Table 1) creating the layered structure (Fig. 2).

### Experimental

The 5 ml of 0.02 *M* chloroform *o*–toluidine solution was poured into the test–tube. The other reactant, 5 ml 0.01 *M* water solution of K_2_PdCl_4_ was carefully added on the top of the organic part. The sealed test–tube with double–layer mixture was put in a dark place. Two weeks later, the yellow plate shape crystals were grown in the chloroform part of the solution.

### Refinement

H atoms bonded to N atoms were located in a difference map. Other H atoms which bonded to C were positioned geometrically and refined using a riding model with C—H = 0.98 Å for CH_3_ with *U*_iso_(H) = 1.5*U*_eq_(C) and C—H = 0.95 Å for CH with *U*_iso_(H) = 1.2*U*_eq_(C)].

### Crystal data

**Table:**

[PdCl_2_(C_7_H_9_N)_2_]	*F*(000) = 392
*M**_r_* = 391.60	*D*_x_ = 1.770 Mg m^−^^3^
Monoclinic, *P*2_1_/*c*	Melting point: 560 K
Hall symbol: -P 2ybc	Mo *K*α radiation, λ = 0.71073 Å
*a* = 12.1841 (3) Å	Cell parameters from 1337 reflections
*b* = 8.0653 (2) Å	θ = 3.0–22.5°
*c* = 7.5407 (2) Å	µ = 1.61 mm^−^^1^
β = 97.346 (2)°	*T* = 173 K
*V* = 734.93 (3) Å^3^	Plate, yellow
*Z* = 2	0.17 × 0.16 × 0.04 mm

### Data collection

**Table:**

Bruker APEXII CCD diffractometer	1502 independent reflections
Radiation source: Fine–focus sealed tube	1118 reflections with *I* > 2σ(*I*)
Graphite	*R*_int_ = 0.074
Detector resolution: 8.26 pixels mm^-1^	θ_max_ = 26.4°, θ_min_ = 1.7°
φ and ω scans	*h* = −15→15
Absorption correction: multi-scan (*SADABS*; Bruker, 2005)	*k* = −10→10
*T*_min_ = 0.777, *T*_max_ = 0.932	*l* = −9→9
8179 measured reflections	

### Refinement

**Table:**

Refinement on *F*^2^	Primary atom site location: Direct
Least-squares matrix: Full	Secondary atom site location: Difmap
*R*[*F*^2^ > 2σ(*F*^2^)] = 0.034	Hydrogen site location: Geom
*wR*(*F*^2^) = 0.060	H atoms treated by a mixture of independent and constrained refinement
*S* = 1.03	*w* = 1/[σ^2^(*F*_o_^2^) + (0.0118*P*)^2^ + 0.6868*P*] where *P* = (*F*_o_^2^ + 2*F*_c_^2^)/3
1502 reflections	(Δ/σ)_max_ < 0.001
97 parameters	Δρ_max_ = 0.48 e Å^−^^3^
2 restraints	Δρ_min_ = −0.66 e Å^−^^3^

### Special details

**Table:**

Geometry. All s.u.'s (except the s.u. in the dihedral angle between two l.s. planes) are estimated using the full covariance matrix. The cell s.u.'s are taken into account individually in the estimation of s.u.'s in distances, angles and torsion angles; correlations between s.u.'s in cell parameters are only used when they are defined by crystal symmetry. An approximate (isotropic) treatment of cell s.u.'s is used for estimating s.u.'s involving l.s. planes.
Refinement. Refinement of *F*^2^ against ALL reflections. The weighted *R*–factor *wR* and goodness of fit *S* are based on *F*^2^, conventional *R*–factors *R* are based on *F*, with *F* set to zero for negative *F*^2^. The threshold expression of *F*^2^ > σ(*F*^2^) is used only for calculating *R*–factors(gt) *etc*. and is not relevant to the choice of reflections for refinement. *R*–factors based on *F*^2^ are statistically about twice as large as those based on *F*, and *R*–factors based on ALL data will be even larger.

### Fractional atomic coordinates and isotropic or equivalent isotropic displacement parameters (Å^2^)

**Table:**

	*x*	*y*	*z*	*U*_iso_*/*U*_eq_	
Pd1	0.0000	0.5000	0.0000	0.01692 (12)	
Cl1	0.06040 (8)	0.77044 (11)	0.02411 (13)	0.0222 (2)	
N1	0.1118 (3)	0.4372 (4)	0.2186 (5)	0.0192 (8)	
H1N	0.130 (3)	0.526 (4)	0.276 (5)	0.024 (12)*	
H2N	0.071 (3)	0.386 (4)	0.294 (5)	0.025 (12)*	
C1	0.2062 (3)	0.3358 (5)	0.1906 (5)	0.0216 (9)	
C2	0.3051 (4)	0.4060 (6)	0.1573 (6)	0.0300 (11)	
C3	0.3912 (3)	0.2990 (6)	0.1258 (6)	0.0330 (11)	
H3	0.4596	0.3446	0.1015	0.040*	
C4	0.3790 (4)	0.1308 (6)	0.1291 (6)	0.0390 (12)	
H4	0.4387	0.0612	0.1073	0.047*	
C5	0.2808 (4)	0.0614 (6)	0.1639 (6)	0.0352 (12)	
H5	0.2722	−0.0556	0.1653	0.042*	
C6	0.1949 (3)	0.1643 (5)	0.1967 (5)	0.0271 (10)	
H6	0.1275	0.1174	0.2238	0.032*	
C7	0.3210 (4)	0.5894 (6)	0.1537 (7)	0.0432 (14)	
H7A	0.2701	0.6373	0.0560	0.065*	
H7B	0.3974	0.6142	0.1350	0.065*	
H7C	0.3060	0.6370	0.2677	0.065*	

### Atomic displacement parameters (Å^2^)

**Table:**

	*U*^11^	*U*^22^	*U*^33^	*U*^12^	*U*^13^	*U*^23^
Pd1	0.0189 (2)	0.0155 (2)	0.0169 (2)	0.0004 (2)	0.00446 (15)	−0.0002 (2)
Cl1	0.0272 (5)	0.0174 (5)	0.0222 (5)	−0.0014 (4)	0.0033 (4)	0.0008 (4)
N1	0.0228 (18)	0.0170 (17)	0.019 (2)	−0.0008 (14)	0.0066 (16)	−0.0040 (16)
C1	0.022 (2)	0.025 (2)	0.017 (2)	0.0002 (17)	0.0021 (18)	0.0009 (17)
C2	0.029 (3)	0.039 (3)	0.022 (3)	0.000 (2)	0.004 (2)	0.000 (2)
C3	0.022 (2)	0.044 (3)	0.033 (3)	0.007 (2)	0.004 (2)	−0.004 (2)
C4	0.034 (3)	0.047 (3)	0.036 (3)	0.014 (2)	0.003 (2)	−0.012 (2)
C5	0.041 (3)	0.030 (2)	0.033 (3)	0.006 (2)	0.000 (2)	−0.005 (2)
C6	0.025 (2)	0.034 (3)	0.022 (3)	0.0000 (19)	0.0007 (19)	0.0015 (19)
C7	0.031 (3)	0.035 (3)	0.063 (4)	0.001 (2)	0.002 (3)	0.005 (3)

### Geometric parameters (Å, °)

**Table:**

Pd1—N1	2.063 (3)	C3—C4	1.365 (6)
Pd1—N1^i^	2.063 (3)	C3—H3	0.9500
Pd1—Cl1^i^	2.3017 (9)	C4—C5	1.376 (6)
Pd1—Cl1	2.3017 (9)	C4—H4	0.9500
N1—C1	1.449 (5)	C5—C6	1.382 (5)
N1—H1N	0.85 (2)	C5—H5	0.9500
N1—H2N	0.90 (2)	C6—H6	0.9500
C1—C2	1.383 (5)	C7—H7A	0.9800
C1—C6	1.392 (5)	C7—H7B	0.9800
C2—C3	1.402 (6)	C7—H7C	0.9800
C2—C7	1.492 (5)		
			
N1—Pd1—N1^i^	180.0	C4—C3—C2	121.5 (4)
N1—Pd1—Cl1^i^	90.13 (10)	C4—C3—H3	119.2
N1^i^—Pd1—Cl1^i^	89.87 (10)	C2—C3—H3	119.2
N1—Pd1—Cl1	89.88 (10)	C3—C4—C5	120.5 (4)
N1^i^—Pd1—Cl1	90.13 (10)	C3—C4—H4	119.8
Cl1^i^—Pd1—Cl1	180.0	C5—C4—H4	119.8
C1—N1—Pd1	118.6 (3)	C4—C5—C6	119.1 (4)
C1—N1—H1N	113 (3)	C4—C5—H5	120.4
Pd1—N1—H1N	108 (3)	C6—C5—H5	120.4
C1—N1—H2N	110 (2)	C5—C6—C1	120.7 (4)
Pd1—N1—H2N	105 (3)	C5—C6—H6	119.7
H1N—N1—H2N	101 (4)	C1—C6—H6	119.7
C2—C1—C6	120.3 (4)	C2—C7—H7A	109.5
C2—C1—N1	121.5 (4)	C2—C7—H7B	109.5
C6—C1—N1	118.2 (4)	H7A—C7—H7B	109.5
C1—C2—C3	117.8 (4)	C2—C7—H7C	109.5
C1—C2—C7	121.8 (4)	H7A—C7—H7C	109.5
C3—C2—C7	120.4 (4)	H7B—C7—H7C	109.5
			
Cl1^i^—Pd1—N1—C1	70.4 (3)	C1—C2—C3—C4	−0.5 (7)
Cl1—Pd1—N1—C1	−109.6 (3)	C7—C2—C3—C4	179.7 (5)
Pd1—N1—C1—C2	90.5 (4)	C2—C3—C4—C5	0.0 (7)
Pd1—N1—C1—C6	−89.0 (4)	C3—C4—C5—C6	−0.5 (7)
C6—C1—C2—C3	1.6 (6)	C4—C5—C6—C1	1.5 (7)
N1—C1—C2—C3	−177.9 (4)	C2—C1—C6—C5	−2.1 (7)
C6—C1—C2—C7	−178.7 (4)	N1—C1—C6—C5	177.4 (4)
N1—C1—C2—C7	1.9 (7)		

Symmetry codes: (i) −*x*, −*y*+1, −*z*.

### Hydrogen-bond geometry (Å, °)

**Table:**

*D*—H···*A*	*D*—H	H···*A*	*D*···*A*	*D*—H···*A*
N1—H1N···Cl1^ii^	0.85 (2)	2.71 (3)	3.410 (4)	141 (3)
N1—H2N···Cl1^iii^	0.90 (2)	2.43 (3)	3.319 (4)	172 (3)

Symmetry codes: (ii) *x*, −*y*+3/2, *z*+1/2; (iii) −*x*, *y*−1/2, −*z*+1/2.
